# β-Human Chorionic Gonadotropin–Secreting Giant Cell Tumor of Bone in the Mandible: Case Report and Comprehensive Literature Review

**DOI:** 10.1007/s12105-025-01883-y

**Published:** 2026-01-13

**Authors:** Andres Flores-Hidalgo, Sholeh Bazrafshan, Hether Khosa

**Affiliations:** 1 Department of Surgery, Department of Pathology and Laboratory Medicine, Division of Oral & Maxillofacial Surgery, University of Cincinnati Medical Center, 200 Albert Sabin Wy, ML 0461, Cincinnati, OH 45219 USA; 2 Department of Pathology and Laboratory Medicine, University of Cincinnati Medical Center, Cincinnati, USA; 3 Department of Surgery, Division of Oral & Maxillofacial Surgery, University of Cincinnati Medical Center, Cincinnati, USA

**Keywords:** Giant cell tumor of bone, Mandible, Β-human chorionic gonadotropin, *H3-3A* mutation, Denosumab

## Abstract

**Introduction:**

Giant cell tumor of bone (GCTB) is a rare but locally aggressive neoplasm which most commonly arises in the epiphyses of long bones of young adults. Occurrence in the mandible is uncommon, and hormone secreting variants are extremely rare.

**Case Presentation:**

We present the case of a 16-year-old female, who presented with progressive swelling and pain in the left mandible after extraction of adjacent teeth. On examination, she had a large ulcerated intraoral mass with marked tooth mobility; imaging revealed a 4.7-cm expansive lytic lesion with cortical erosions and displacement of the inferior alveolar canal. Histopathologic evaluation revealed sheets of mononuclear stromal cells interspersed with numerous osteoclast-like giant cells. Immunohistochemical stains showed strong positivity for H3G34W and p63. Genomic sequencing confirmed the presence of an H3-3A p.G35W hotspot mutation and also identified a pathogenic FANCA p.R951Q variant. Investigations prior to treatment showed an elevated serum β-human chorionic gonadotropin (β-hCG) level despite the patient being negative for pregnancy status. Following this the patient began denosumab therapy, which was associated clinical improvement, plus a decline in β-hCG, confirming the tumor as the source of hormone secretion.

**Discussion:**

There is only one other case that has been documented of a β-hCG–producing GCTBoccurring in the base of the skull with secondary aneurysmal bone cyst–like changes.This appears to be the first reported instance of β-hCG–secreting GCTB in the gnathicbones. This case exemplifies the diagnostic challenges of rare presentations of GCTBand alerts clinicians to the potential misleading presentation of hormone expression.

## Introduction

Giant cell tumor of bone (GCTB) is a typically benign, but locally aggressive tumor characterized by the proliferation of mononuclear stromal cells with multiples osteoclast-like multinucleated giant cells. GCTB affects skeletally mature individuals between the ages of 20 and 40, and has a slight female predominance [1]. Although, GCTB generally arises in the epiphyseal regions of long bones (most commonly distal femur, proximal tibia, or distal radius), it may occur in virtually any region of the skeleton [2]. In recent years, advances in molecular pathology have demonstrated also that most conventional GCTB have mutations in the *H3F3A* gene, most often the G34W substitution that not only aids in tumor formation, but is also an essential diagnostic marker of GCTB [3, 4].

Although benign histologically, giant cell tumor of bone (GCTB) is notorious for its local recurrence, with some studies reporting rates of recurrence as high as 50% following curettage, and infrequently, metastasis to the lungs occurs. This report describes the case of a 16-year-old healthy female patient with a GCTB in the left mandible and details the clinical presentation, diagnosis, and significant histopathological and molecular findings [5].

## Case Report

### Clinical and Radiographic Presentation

A 16-year-old female was referred by an outside general dentist for evaluation and management of an expansile lesion to the left mandible. The patient had some swelling and discomfort a few weeks prior to her consultation. She obtained a second opinion from another dental provider who suspected it was a serious infectious process, and recommended tooth extractions and antibiotics. After extraction the patient felt her swelling and pain progressively increased. She was referred to the UC Health Medical Center, Division of Oral & Maxillofacial Surgery, for management.

**Fig. 1:**
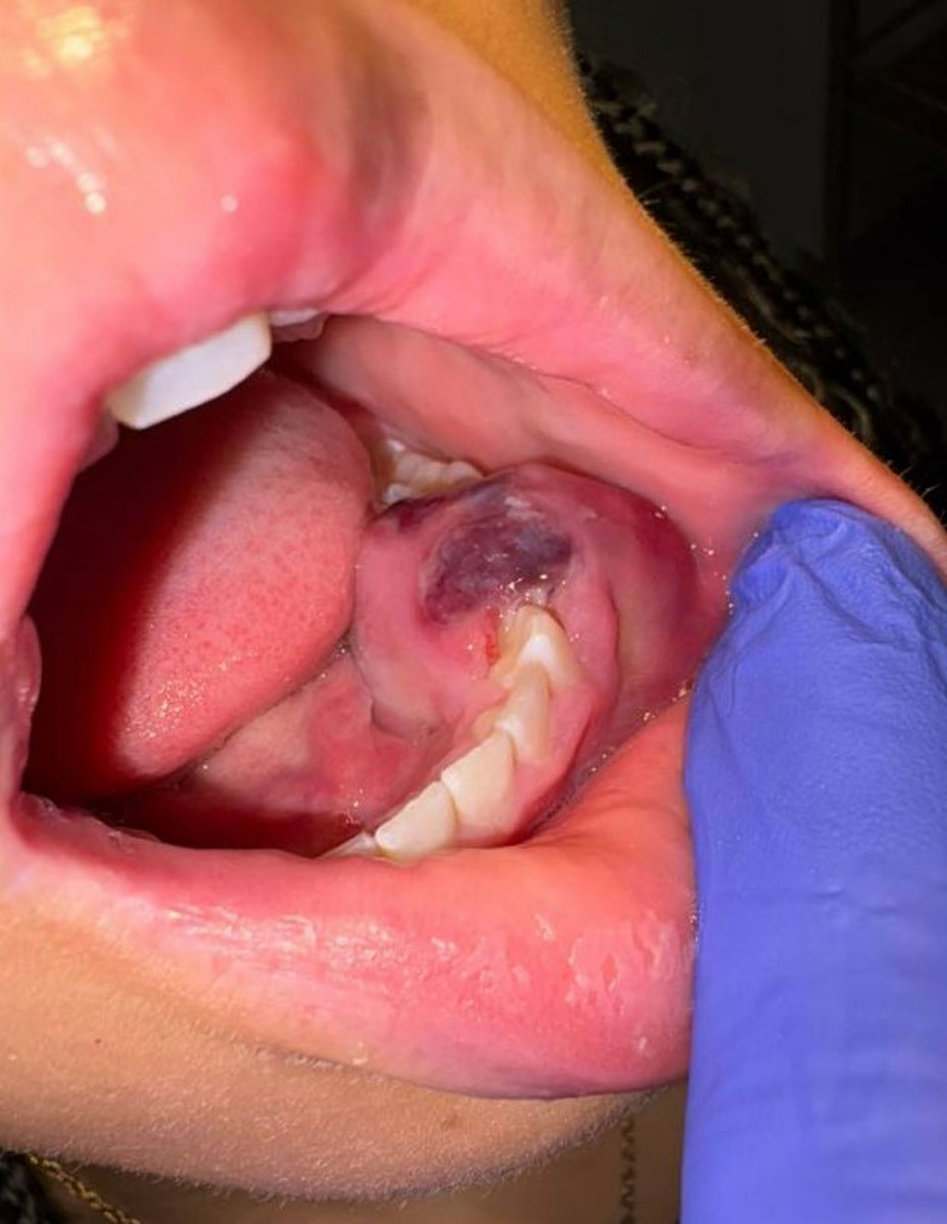
Intraoral presentation of the lesion. Large, ulcerated mass on the left mandible

**Fig. 2:**
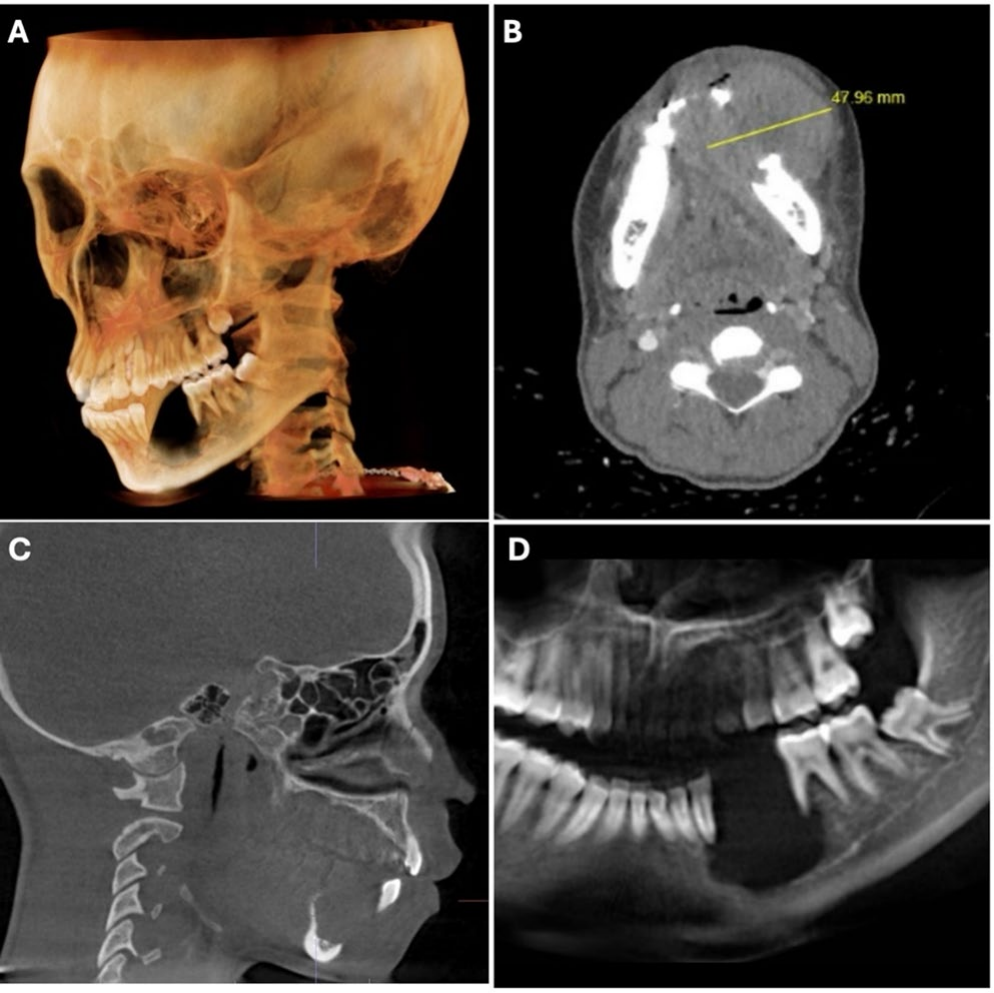
Radiographic presentation. **A** 3D rendition of Cone Beam Computerized tomography (CBCT). **B** Transverse view with measurement of the diameter of the lesion. **C** Sagittal view. **D** Portion of panoramic radiograph

The patient had no significant medical history and not known drug allergies. Clinical examination revealed unilateral enlargement of the lower third of the face. The intraoral examination revealed a large, ulcerated mass on the right mandible, premolar area (Fig. 1). Teeth #20 and 21 are absent and adjacent #22 and #30 show class IV mobility. A scanned Cone Beam computerized tomography (CBCT) taken of the area of interest revealed a large radiolucency, of ~ 4.7 cm in diameter, in the left mandible around teeth #20 and 21, to include inferior displacement of the inferior alveolar nerve. Erosion of the buccal and lingual cortex is evident (Fig. 2).

## Histopathological Findings

**Fig. 3:**
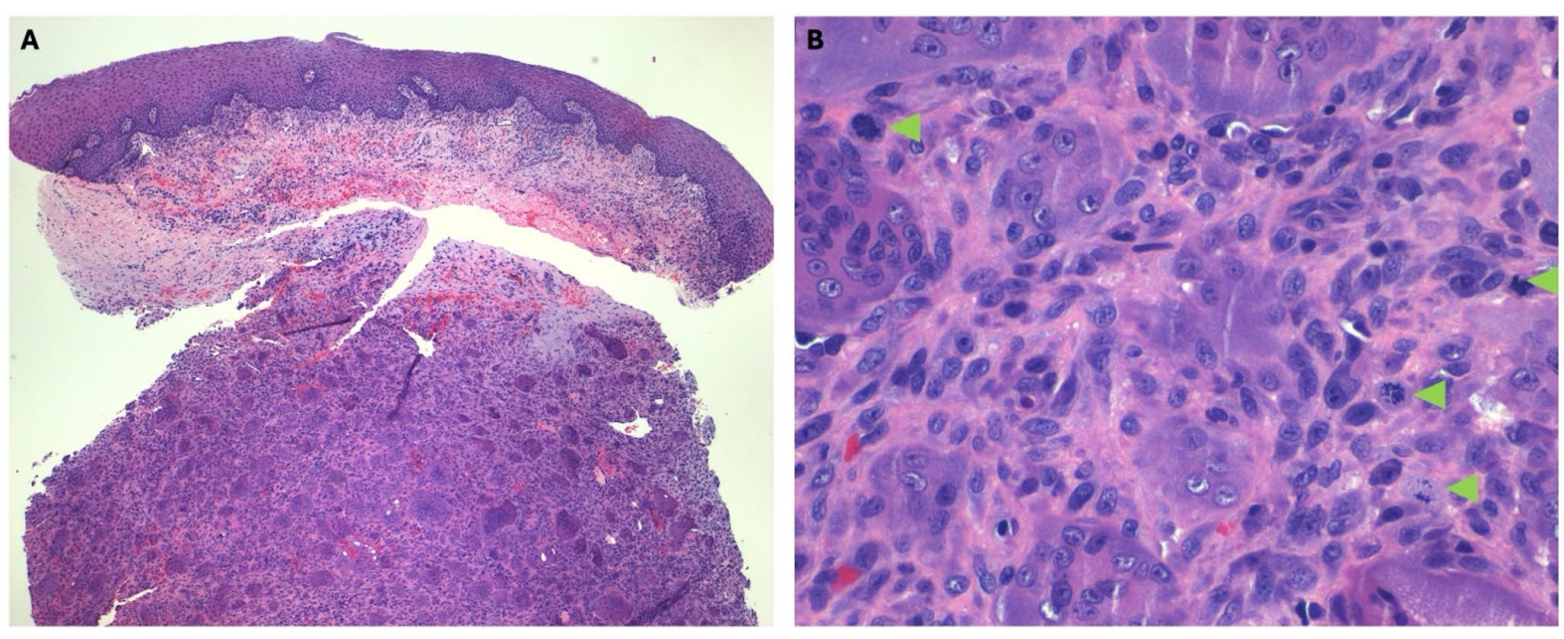
Microcopy of incisional biopsy of the lesion. **A** Osteoclast-like multinucleated giant cells embedded in sheets of a mononuclear cell population underlying the surface oral mucosa [Hematoxylin and Eosin – 100X magnification]. **B** Higher power of magnification shows the multinucleated giant cells intermixed with a sheet of spindle cell mononuclear cells and scattered mitotic figures (green arrowheads) [Hematoxylin and Eosin – 400X magnification]

Based on the clinical and radiological presentation, an incisional biopsy was performed on the same visit, under local anesthetic. A 2 cm x 2 cm piece of tissue was excised and sent for permanent histopathology. The microscopic findings showed a very cellular lesion with large, osteoclast-like, multinucleated giant cells intermixed with sheets of round to spindle mononuclear cells with pale eosinophilic cytoplasm, nuclei with dispersed chromatin and small nucleoli (Fig. 3a). Mitotic figures were also observed throughout the specimen (Fig. 3b), predominantly fewer than five per ten high-power fields. Hemorrhage, hemosiderin and foamy macrophages were seen too.

**Fig. 4:**
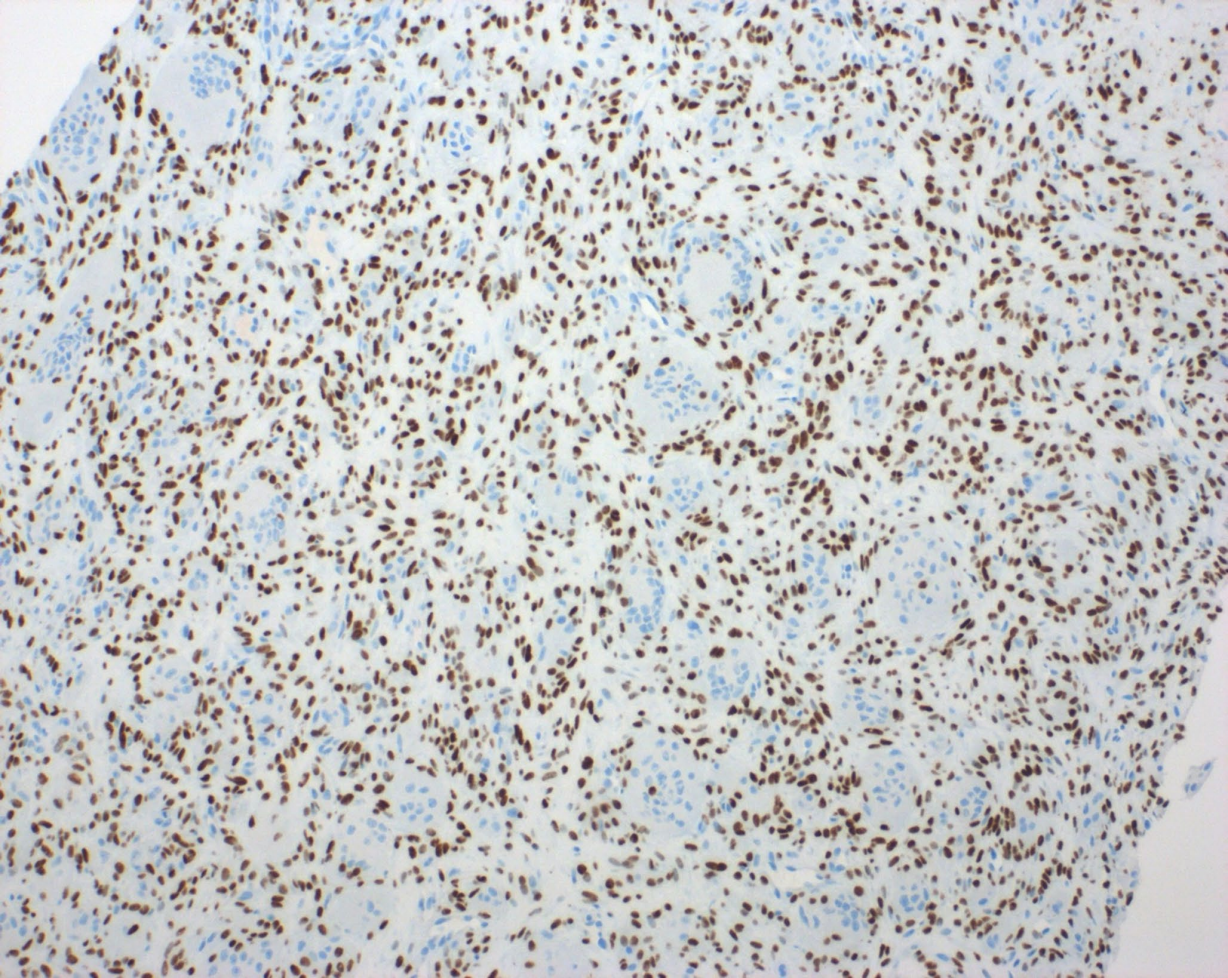
H3G34W immunohistochemical stain was performed showed strong nuclear reactivity in the neoplastic mononuclear cells sparing multinucleated giant cells [200X magnification]

**Fig. 5:**
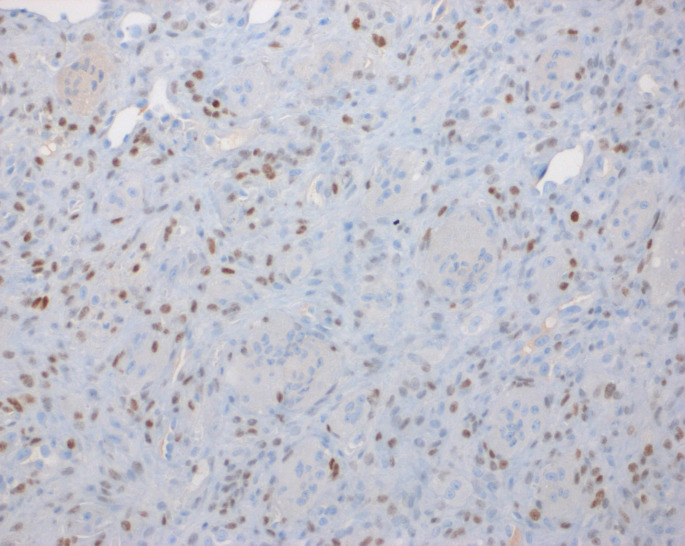
p63 immunohistochemical stain shows a similar staining pattern as H3G34W [200X magnification]

An initial diagnosis of giant cell granuloma (CGCG) was considered, but because of the aggressive behavior of the tumor and microscopic features, a H3G34W immunohistochemistry (IHC) was performed which showed strong nuclear reactivity in the neoplastic mononuclear cells of the tumor sparing multinucleated giant cells (Fig. 4). The p63 stain was also positive in the neoplastic population, though with a similar pattern to the stain to H3G34W (Fig. 5). From a clinical, microscopic, immunohistochemical, and molecular perspective a diagnosis of giant cell tumor of bone was made.

## Molecular Analysis

Cincinnati Children’s Cancer Sequencing (CinCSeq) Comprehensive Panel was performed and reported a homozygous hotspot *H3-3 A* p.G35W mutation consistent with histopathologic findings of specimen and rendered diagnosis. An additional pathogenic *FANCA* p.R951Q variant was found at variant allele frequency ~ 50%, which raised the possibility of germline. Tumor mutational burden was 3 mut/Mb. A complete listing of the identified DNA variants is found in Table 1.

## Management

Pre-treatment assessment indicated a positive urine pregnancy test and β-human chorionic gonadotropin (β-hCG) serum level of 5.1 mIU/mL, but pregnancy had been definitively excluded. By the time of manuscript submission, the patient remained on active treatment. She was at Cycle 1, Day 21 of denosumab therapy (with plan of four weeks of weekly therapy transitioning to monthly maintenance dosing). At the last follow-up appointment, the tumor showed measurable decrease in size and the patient showed improvement in oral intake. Additionally, the β-hCG level had decreased to 2.6 mIU/mL, thus supporting its association with the neoplasm.

## Discussion

Giant cell tumor of bone is an overall benign but locally aggressive neoplasm, which is defined as the proliferation of mononuclear stromal cells with multinucleated osteoclast-like giant cells scattered within the neoplasm. They are considered histologically benign, but GCTB has a marked tendency toward local recurrence, and in rare cases may metastasize, mostly to the lungs [1, 5].

GCTB accounts for approximately 4–5% of all primary bone tumors and 20% of all benign bone tumors [6, 7]. The principal demographic affected by GCTB is young adults between 20 and 40 years of age, with a slight female predominance, occurring primarily in the epiphyseal region of long bones - most commonly around the knee; GCTB can also occur in the distal radius and sacrum [8].

There is a clear link in the pathogenesis of GCTB to the *H3-3 A* gene changes, which encodes histone H3.3. The vast majority (approximately 92%) of all GCTBs have a mutation in codon 34, usually the p.Gly34Trp (G34W) [3, 4]. This mutation only happens in the neoplastic mononuclear stroma cells that are believed to be the neoplastic component of GCTB. The *G34W* mutation is believed to affect histone methylation patterns, thus modifying transcription and causing RANKL overexpression to promote osteoclastogenesis. The multinucleated giant cells are believed to be reactive from the monocyte–macrophage lineage cells, which arose from this signaling [9]. The *H3-3 A* gene that encodes the histone protein H3.3 acts as an oncogenic driver for developmental and transcriptional mechanisms. Canonical hotspot mutations arise at lysine 28 and glycine 34, with the G34 codon, specifically, the G34W substitution having the strongest correlation for giant cell tumor of bone [3].

There are also rare mutations such as the *H3F3B* alterations that have been reported, however, they are generally not related and may be related to specific anatomic locations [10]. The *H3-3 A* mutation has clinical significance since it is absent in histologic mimics such as aneurysmal bone cyst and chondroblastoma. Additionally, Gomes et al. 2014, found that *H3-3 A* p.Gly34 TRP, was not present in aggressive CGCG of the jaw and giant cell lesions of patients with cherubism [11].

Through next generation sequencing we also identified a pathogenic *FANCA* p.R951 variant. *FANCA* is a tumor suppressor gene that encodes the protein Fanconi anemia complementation group A, which is a member of the Fanconi anemia core complex that assembles at damaged chromatin and activates the DNA response by coordinating BRCA 1/2 proteins. Mutations of *FANCA* are the most prevalent, making up 65% of the heritable Fanconi’s anemia cases that are a cancer predisposition syndrome with elevated risk for a variety of malignant neoplasms [12].

GCTB consists histologically of three main cell types. The main cells are mononuclear stromal cells, the neoplastic cell population; these appear as round to oval cells with vesicular nuclei, moderate eosinophilic cytoplasm, and the occasional nucleolus. Multinucleated osteoclast-like giant cells, which can contain 20 to 50 + nuclei, are spaced randomly throughout the lesion and often clustered around hemorrhage. Finally, there are mononuclear histiocytic cells which are part of the reactive component and tend to be fewer than the stromal cells [13]. Mitotic activity is noted but atypical mitotic figures are infrequent in conventional GCTB. Areas of hemorrhage, hemosiderin deposition, and necrosis are common. Secondary aneurysmal bone cyst–like changes may be present in ~ 14% of cases [14]. Imunohistochemically the mononuclear stromal cells demonstrate strong nuclear positivity for H3.3 G34W mutation-specific antibody, whereas the giant cells are positive for CD68 [13, 15, 16].

Typical radiographic features include an eccentric lytic lesion affecting the epiphysis and extending to the subchondral bone with non-sclerotic margins, the lack of matrix mineralization, and an appearance similar to the radiographic presentation in our case [17]^,^ [18].

Although histologically benign, GCTB has a 20–50% local recurrence rate depending on surgical management [19]. Malignant giant cell tumor of bone is an uncommon aggressive variant that accounts for less than 5% of all GCTB. It can arise de novo (primary malignant GCTB) or more commonly as a secondary transformation from a previously established benign GCTB, generally after multiple recurrences or following prior radiotherapy [20]. Malignant transformation is very rare, typically occurs after radiation therapy, or with recurrent disease. The prognostic factors for recurrence include the site (distal radius and sacrum), inadequate surgical margins, and secondary aneurysmal bone cyst changes [21, 22].

β-human chorionic gonadotropin (β-hCG)–expressing giant cell tumor of bone (GCTB) is incredibly rare. Although the production of β-hCG has been defined for some bone sarcomas, for example, osteosarcoma [23]. However, β-hCG expression in GCTB, in gnathic bones, is exceedingly rare. To date, the only case found in the medical literature described the finding of a GCTB that had changes of an aneurysmal bone cyst-like changes in the head and neck region. In this case, the patient’s serum β-hCG levels returned to normal following resection, confirming that the tumor was the source of hormone production [24]. In our patient, the levels of β-hCG dropped with treatment with denosumab. To the best of our knowledge, this may be the first reported case of β-hCG secreting GCTB in the mandible. Yet, Lawless et al. (2014) published an institutional case series of 40 patients with β-hCG–secreting GCTB [23]. In one case, the tumor arose in the posterior mandible and showed mild positivity for β-hCG on IHC. However, the authors did not clarify whether this patient had elevated serum β-hCG levels and noted that, in several cases, there was a discrepancy between immunohistochemical expression and serum findings. A full summary of reported GCTB in the head and neck region is presented in Table 2.

There is a gap in the literature regarding the prognostic and therapeutic implications of β-hCG expression in GCTB. No systematic studies have been published that investigate the impact of β-hCG positivity on recurrence risk, metastatic risk, or therapy response in these tumors [24–26]. Therefore, more research is needed to investigate whether β-hCG expression has clinical relevance other than causing challenges in the diagnostic interpretation. The current standard of care for GCTB remains surgical curettage, either with or without local adjuvant therapy, and denosumab for unresectable or recurrent disease [25, 27]. Though β-hCG expression does not currently influence best treatment practices, recognition of this rare finding is important to ensure the correct diagnosis and highest quality clinical care [23, 24].

Complete surgical excision is the gold standard for GCTB in the mandible, ideally with negative margins to minimize chances of recurrence [27, 28]. Extended intralesional curettage with local adjuvants is preferred, where possible, with recurrence rates up to 27%, depending on technique and adjuvant used. En bloc resection may be required for large or recurrent lesions, but must be weighed against mandibular reconstruction morbidity, particularly in younger patients such as ours [27, 28].

The use of denosumab, a monoclonal antibody to RANKL has changed the management of unresectable or recurrent GCTB^28^. Denosumab reduces the number of giant cells and induces bone formation. While long-term follow up and recurrence rates after discontinuation remain under assessment [9, 16].

## Conclusion

In conclusion, GCTB is rare in the gnathic bones and represents a small percentage of all documented cases. Our case of mandibular GCTB in an otherwise healthy 16-year-old female demonstrates the importance of ruling out GCTB when examining head and neck giant cells lesions, such as CGCG. While β-hCG expression does not presently impact established treatment protocols, its recognition is critical for accurate diagnosis, particularly those occurring in atypical locations and in atypical age groups or in patients, and the provision of the highest standard of clinical care.

The diagnosis requires the careful integration of clinical, radiographic, and histopathologic features, as well as molecular confirmation such as *H3-3 A* mutation testing. Although surgical resection remains the standard of care, the unique anatomy and functional role of the mandible require a thoughtful and individualized approach to minimize disease while maximizing esthetics and maintaining oral function.

Though the prognosis is usually reasonable following appropriate management, the risk of local recurrence, especially in younger patients, warrants long-term follow-up.

**Table 1:** Detailed results of DNA variant encountered by next generation sequencing

Biomarker	Variant		VAF/CNR (%)	Location	Classification
*DNA variants*					
*H3-3 A*	p.G35W	c.103G > T	27	exon-2	Tier 1/2
*FANCA*	p.R951Q	c.2852G > A	50	exon-29	Tier 1/2
*ERCC2*	p.L152P	c.455T > C	49	exon-6	VUS
*PRKDC*	p.I3193M	c.9579 A > G	47	exon-69	VUS
*TEK*	p.T693I	c.2078 C > T	45	exon-13	VUS
*MSH6*	p.?	c.4001 + 10_4001 + 11insAACTA	45	intron-9	VUS
*KMT2C*	p.S207C	c.620 C > G	45	exon-5	VUS
*BCORL1*	p.A130T	c.388G > A	45	exon-4	VUS
*CSF1R*	p.T467M	c.1400 C > T	44	exon-9	VUS
*BCOR*	p.P1156T	c.3466 C > A	32	exon-7	VUS
*FGFR1*	p.G348_S353del	c.1043_1060del	26	exon-8	VUS
*SLX4*	p.P1317_P1321del	c.3949_3963del	5	exon-12	VUS
*Fusions/SV*					
None					
*Copy number variations*					
None					

**Table 2:** This table summarizes the clinical, pathological, and diagnostic features of reported cases of β-hCG secreting giant cell tumor of bone in the head and neck region, highlighting the extreme rarity of β-hCG production in this tumor type and anatomical location, as documented in the medical literature

Case/Series	Location	β-hCG Secretion	Key findings	References
Fitzhugh et al. [24]	Sphenoid bone (skull base)	Yes	β-hCG detected in serum and tumor; resolved after resection	[24]
Zhang et al. (2013)	Skull (18 cases)	No	No β-hCG secretion reported	[29]
Freeman et al. (2016)	Skull base (meta-analysis)	No	No β-hCG secretion reported	[30]
Gao et al. [28]	Maxillofacial region	No	No β-hCG secretion reported	[28]
Battoo et al. (2012)	Nasal cavity/skull base	No	Oncogenic osteomalacia, no β-hCG secretion	[31]
Akyigit et al. (2014)	Temporal bone/TMJ	No	No β-hCG secretion reported	[32]
Inoue et al. (2016)	Clivus	No	No β-hCG secretion reported	[33]

## Data Availability

No datasets were generated or analysed during the current study.
